# Biophysical and structural analyses of the interaction between the SHANK1 PDZ domain and an internal SLiM

**DOI:** 10.1042/BCJ20240126

**Published:** 2024-07-10

**Authors:** Yue Li, Chi H. Trinh, Amanda Acevedo-Jake, Diana Gimenez, Stuart L. Warriner, Andrew J. Wilson

**Affiliations:** 1 School of Chemistry, University of Leeds, Woodhouse Lane, Leeds LS2 9JT, U.K.; 2 Astbury Centre for Structural Molecular Biology, University of Leeds, Woodhouse Lane, Leeds LS2 9JT, U.K.; 3 School of Molecular and Cellular Biology, University of Leeds, Woodhouse Lane, Leeds LS2 9JT, U.K.; 4 School of Chemistry, University of Birmingham, Edgbaston, Birmingham B15 2TT, U.K.

**Keywords:** PDZ domains, peptide interacting motifs, protein–protein interactions

## Abstract

The PDZ (Postsynaptic density protein-95[PSD-95]/Discs-large) domain, prevalent as a recognition module, has attracted significant attention given its ability to specifically recognize ligands with consensus motifs (also termed PDZ binding motifs [PBMs]). PBMs typically bear a *C*-terminal carboxylate as a recognition handle and have been extensively characterized, whilst internal ligands are less well known. Here we characterize a short linear motif (SLiM) — EESTSFQGP — as an internal PBM based on its strong binding affinity towards the SHANK1 PDZ domain (SHANK1_656–762_ hereafter referred to as SHANK1). Using the acetylated analogue Ac-EESTSFQGP-CONH_2_ as a competitor for the interaction of SHANK1 with FAM-Ahx-EESTSFQGP-CONH_2_ or a typical fluorophore-labelled *C*-terminal PBM — *GKAP* — FITC-Ahx-EAQTRL-COOH — the internal SLiM was demonstrated to show comparable low-micromolar IC_50_ by competition fluorescent anisotropy. To gain further insight into the internal ligand interaction at the molecular level, we obtained the X-ray co-crystal structure of the Ac-EESTSFQGP-CONH_2_/SHANK1 complex and compared this to the Ac-EAQTRL-COOH/SHANK1 complex. The crystallographic studies reveal that the SHANK1 backbones for the two interactions overlap significantly. The main structural differences were shown to result from the flexible loops which reorganize to accommodate the two PBMs with distinct lengths and terminal groups. In addition, the two *C*-terminal residues Gly and Pro in Ac-EESTSFQGP-CONH_2_ were shown not to participate in interaction with the target protein, implying further truncation and structural modification using peptidomimetic approaches on this sequence may be feasible. Taken together, the SLiM Ac-EESTSFQGP-CONH_2_ holds potential as an internal ligand for targeting SHANK1.

## Introduction

**Figure 1.**
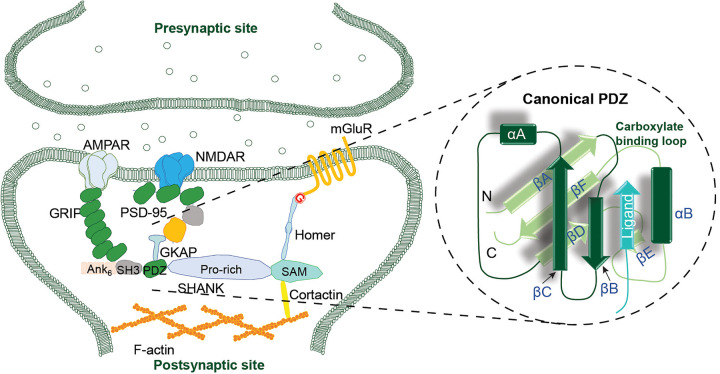
Role of PDZ interactions in synaptic function. Schematic illustrating the scaffolding role of SHANK protein at the postsynaptic site of nerve cells. The expansion illustrates the canonical structure of a typical PDZ domain, consisting of two α helices and six β-strands forming a sandwich-like configuration onto which the PBM can dock via β-strand complementation.

The *SHANK* (SH3 and multiple ankyrin repeat domain) protein acts as a scaffolding protein located at excitatory glutamatergic synapses (Figure 1); it plays a crucial role in synapse formation, maintenance, function, and development [1]. As one of the key components of *SHANK* structures, PDZ [Postsynaptic density protein-95(PSD-95)/Discs-large (DLG)/zonula occludens-1(ZO1)] domains are a large class of protein–protein interaction (PPI) modules that are widely conserved from yeast to humans [2,3]. Moreover, PDZ domains play a significant role in modulating intracellular communication networks specifically and efficiently, such as trafficking, recruiting, and assembly of intracellular enzymes and membrane receptors into signal-transduction complexes [2,3].

According to an NMR and X-ray crystallography analysis, over 200 PDZ structures have been characterized to provide extensive information on the molecular mechanism by which PPIs of PDZ domains regulate biological function [4]. A canonical PDZ domain (Figure 1) is usually composed of roughly 90 amino acid residues, consisting of 5–6 β-strands (βA-βF) capped by a short helix αA and a long α-helix αB, where the *N*- and *C*-terminal strands βA and βF are in mutual proximity [5]. This β-sandwich-like conformation mediates recognition and interaction of proteins bearing PDZ binding motifs (PBMs). Significantly, PDZs possess a highly conserved carboxylate-binding loop formed by the second α-helix (αB) and the second β-strand (βB) in an antiparallel fashion; discoveries and characterization of PBMs have established that *C*-terminal PBMs predominate [6]. For instance, studies on the interaction of the SHANK1-PDZ protein with the pentapeptide _642_DETNL_646_ from *β*-PIX (ARHGEF7) [7] or the hexapeptide _661_EAQTRL_666_ from *GKAP* [8], have shown that both bind the target protein with a similar binding mode according to crystallographic analyses (RMSD of 0.69 Å) [9]. The consensus motif T-x-L-COOH (x can be any amino acid) in the two *C*-terminal PBMs makes extensive contacts with the target PDZ backbones αB and βB, where leucine is inserted into the carboxylate binding loop and threonine makes a conserved hydrogen bond to a SHANK1-PDZ histidine [9]. This is consistent with the canonical class I mode of interaction and therefore the two residues are referred to as Leu (0) and Thr (-2) [5]. A further example of class I mode of interaction is the PSD-95-PDZ1 and PDZ2 domains which recognize the *C*-terminal consensus motif T/S-X(D/E/A)-V/I-COOH of the Shaker-type K^+^ channels or the NMDAR2 (*N*-methyl-d-aspartate receptors) [10]. Due to a preference for Val/Ile or Leu at position 0 of the PBM consensus motif, PSD-95-PDZ and SHANK1-PDZ exhibit distinct affinities towards identical *C*-terminal ligands, as emphasized in our previous work where *GKAP* and peptide-fragment hybrids were shown to bind with lower affinity to PSD-95-PDZ domain than to the SHANK1-PDZ domain [11].

It has also been established that some PDZ domains can in addition to classical *C*-terminal PBMs, recognize internal sequences which do not involve a conserved carboxylate group at the *C*-terminus [12,13]. Moreover, it has been shown that recognition of PBMs may exploit selection from a dynamic ensemble of conformers [14]. These data emphasized the plasticity of PDZ/PBM interactions. Moreover, internal ligands can be found in both structured regions [15] and intrinsically disordered regions bearing short linear motifs (SLiMs) [16]. Given that SLiMs can exert functions on their binders independently of the full-context of the parent proteins [17], internal ligands may constitute alternative templates for the design of ligands that modulate target PDZ domains.

Recently phage display was used to identify a series of internal ligands — from the human proteome — targeting the SHANK1-PDZ domain with a short consensus motif, x-T-x-F-x (x can be any amino acid) with low to moderate micromolar binding affinities towards SHANK1-PDZ [18]. A crystal structure was determined for a fusion protein comprising the SHANK1-PDZ domain attached *C*-terminally to a 16-mer internal ligand derived from *ARAP3* [3.0 (±0.3) μM] [19]. Informed by these results, in the present work we selected three of these internal sequences — including the dominant sequence identified through position-specific scoring matrices (PSSMs) representing the binding enriched sequence from ProP-PD — and titrated their fluorophore-labelled analogues against the target protein SHANK1-PDZ domain (SHANK1_656–762_ hereafter referred to as SHANK1). After identifying FAM-Ahx-EESTSFQGP-CONH_2_ as the internal PBM with most promising potency, the acetylated analogue Ac-EESTSFQGP CONH_2_ was confirmed to exhibit comparable SHANK1 potency to a *C*-terminal PBM — *GKAP* — Ac-EAQTRL-COOH — and bind at the same site using fluorescent anisotropy (FA) direct and competition assays. ITC experiments and variable temperature (VT) FA analyses provided thermodynamic insight into the interaction. We obtained an X-ray co-crystal structure of the SHANK1/Ac-EESTSFQGP-CONH_2_ complex; comparison with SHANK1/Ac-EAQTRL-COOH revealed that the PDZ backbones are similar to each other suggesting large conformational differences are not required to accommodate internal PBMs and instead that local differences in flexible loops are harnessed to accommodate the two different ligand classes. Overall, the present work identified the SLiM Ac-EESTSFQGP-CONH_2_ as an alternative SHANK1 internal ligand with good potency to the *C*-terminal ligand SHANK1/Ac-EAQTRL-COOH complex.

## Results and discussion

### An internal PBM exhibits µM SHANK1 binding potency

**Figure 2.**
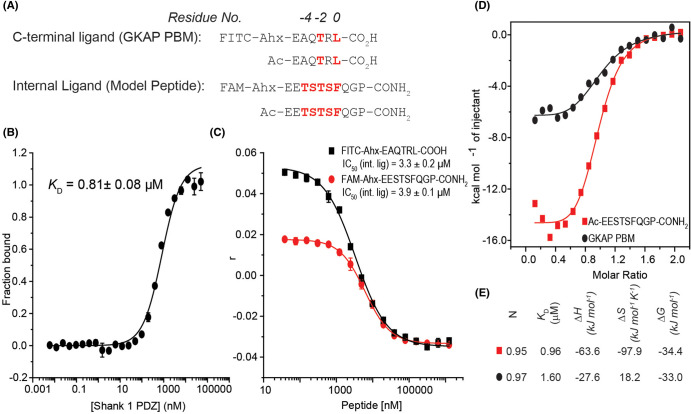
Biophysical characterization of Internal PBM/SHANK1 interaction. (**A**) *N*-terminally acetylated and fluorophore-labelled amino acid sequences of the representative *C*-terminal ligand GKAP PBM and internal ligands; according to the classification of the SHANK1 and its PBM consensus motif, residues Leu in GKAP PBM and Phe in the model peptide are annotated as position 0; residues Thr in both PBMs are annotated as position -2; (**B**) fluorescent anisotropy direct titration curve for FAM-Ahx-EESTSFQGP-CONH_2_/SHANK1 (50 mM NH_4_OAc, pH 6.5 buffer); and, (**C**) fluorescence anisotropy competition assays for Ac-EESTSFQGP-CONH_2_ (50 mM NH_4_OAc, pH 6.5 buffer using 50 nM FITC-Ahx-EAQTRL-COOH (black) and FAM-Ahx-EESTSFQGP-CONH_2_ (red) as tracers); (**D**) fitted thermograms measured by ITC; and, (E) thermodynamic signature for the tested peptides Ac-EESTSFQGP-NH_2_ and Ac-EAQTRL-COOH (data were acquired in 25 mM Tris, pH 7.5 containing 150 mM NaCl at 25°C by injecting 500 μM peptide solution into 50 μM protein in the cell).

According to a recent report on SHANK1-PDZ ligands [18], a series of *C*-terminal and internal PBMs with low to moderate micromolar binding affinities were identified using proteomic peptide-phage display. Based on this research, we selected three sequences: ARAP3_1414–1429_, ELFN1_579–594_ and a 9-mer PSSMs-generated sequence EESTSFQGP (Supplementary Table S1 and Figure 2A) as internal ligand models for initial screening. Their *C*-terminal amidated analogues with *N*-terminal fluorophore conjugation were synthesized and tested individually using direct and competition FA titrations. The short sequence: FAM-Ahx-EESTSFQGP-NH_2_ exhibited the most favourable binding affinity towards SHANK1 with a *K*_D_ of 0.81 ± 0.08 μM (Figure 2B and Supplementary Figure S1) whilst its acetylated analogue Ac-EESTSFQGP-NH_2_ exhibited the highest inhibitory potency (IC_50_ = 3.9 ± 0.1 μM) when titrated against the SHANK1-PDZ/FAM-Ahx-EESTSFQGP-NH_2_ interaction (Figure 2C). The latter peptide was further used to compete against a fluorophore-labelled *C*-terminal *GKAP* PBM — FITC-Ahx-EAQTRL-COOH — for binding to SHANK1. The two competition assay results (Figure 2C) indicate Ac-EESTSFQGP-NH_2_ inhibits both interactions with similar low-micromolar potency, suggesting the internal ligand Ac-EESTSFQGP-NH_2_ is comparable in affinity to the *C*-terminal PBM and competes for a similar site on SHANK1. Taken together, the SLiM peptide Ac-EESTSFQGP-NH_2_ was chosen as the internal ligand model for further, more detailed characterization.

### Ac-EESTSFQGP-NH_2_ and *GKAP* PBM exhibit opposing thermodynamic signatures for binding to SHANK1 PDZ as determined by ITC

We next used isothermal titration calorimetry system to obtain thermodynamic parameters for binding of both peptide ligands to SHANK1 (Figure 2D). In line with the FA results, Ac-EESTSFQGP-NH_2_ and Ac-EAQTRL-COOH bound to the target protein with comparable affinities in 1:1 stoichiometries. Ac-EESTSFQGP-NH_2_ showed a more favourable enthalpic contribution but higher entropic cost of binding to SHANK1 in comparison with Ac-EAQTRL-COOH (Figure 2E). We performed further VT FA experiments and van't Hoff analyses to explore the role of buffer (HEPES, phosphate, MOPS with different hydrophobicity's), salt and pH on the interaction of the C-terminal and internal ligand with SHANK1 (Supplementary Figure S2). Similar thermodynamic signatures were obtained for the ITC experiments (acetylated peptides) and FA experiments (labelled peptides). The difference in binding affinities (ΔΔ*G*) between the *C*-terminal and internal ligand show only subtle variation across different buffers (∼1 kJ mol^−1^) but greater variation with/without salt, indicating the enthalpic increase in binding of the internal ligand to SHANK1 is associated with changes in backbone or side chain interactions between ligand and protein which, become shielded with increasing salt concentration. The similar ΔΔ*G* values when pH is varied indicate that proton transfer is not a key factor in either interaction. Crucially the overall thermodynamic signature is consistent across the varied conditions, i.e. more favourable enthalpy, but less favourable entropy for the internal ligand in comparison with the C-terminal ligand; this supports a hypothesis that differences in entropy of binding are associated with structural differences in the interaction of the two ligands with SHANK1 (*vida infra*). We also carried out thermal melting analyses of SHANK1 in the presence and absence of Ac-EESTSFQGP-NH_2_ and Ac-EAQTRL-COOH using circular dichroism (Supplementary Figure S3). Both ligands stabilize SHANK1 against unfolding (∼3°C to 4°C) with the C-terminal ligand having a slightly more pronounced effect. Although these differences in *T_m_* are small, such variations are often used to inform fragment elaboration screening campaigns [20]; the data indicate that ligand binding stabilizes SHANK1 to different extents, which may be associated with adaptation of the PDZ domain to the different ligands.

### The SHANK1/Ac-EESTSFQGP-NH_2_ co-crystal structure illustrates that loop flexibility is key in accommodating the internal PBM

**Figure 3.**
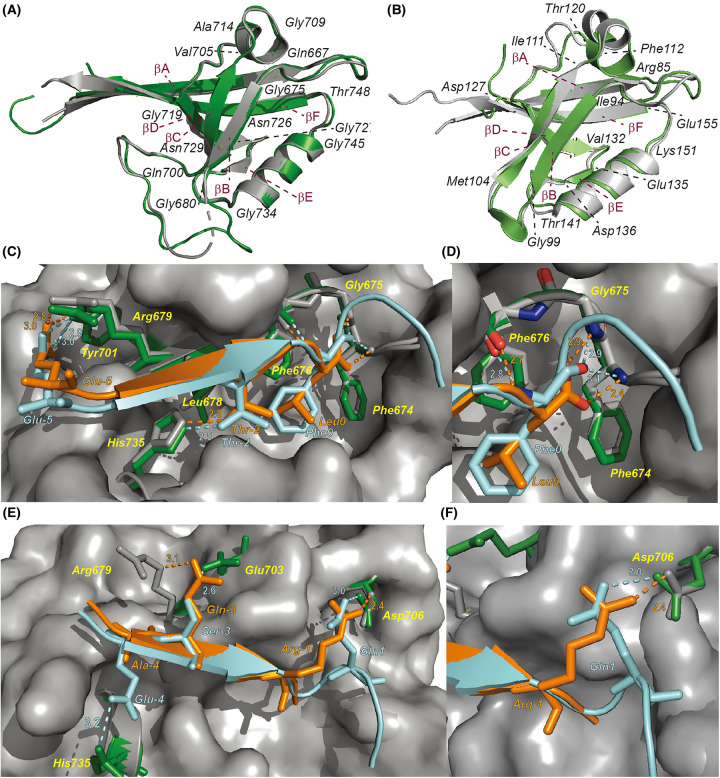
Crystallographic analyses of the SHANK1/Ac-EESTSFQGP-CONH_2_ complex. (**A**) Superimposed crystal structures of SHANK1/*GKAP* PBM (grey, PDB code: 1Q3P) and SHANK1/Ac-EESTSFQGP-CONH_2_ (forest green, PDB code: 8S1R) with a full-length calculated RMSD of 0.45 Å (PBMS not shown). (**B**) Superimposed crystal structures of Syntrophin PDZ/TRPV3 (light grey, PDB code7QQN) and Syntrophin PDZ/nNOS (light green, PDB code: 1QAV) with a full-length calculated RMSD of 0.79 Å (PBMS not shown). (**C**) Superimposed co-crystal structures of SHANK1/Ac-EESTSFQGP-CONH_2_ (cyan) PPI and SHANK1/Ac-EAQTRL-OH (orange) PPI. Both residues at P-2, P-5 and P0 interact with the same target residues, (**D**) magnified figure showing subtle atomic differences on the Leu(0) and Phe(0) interactions with backbone residues F674, G675 and F676; (**E**) Residues at P-4, P-3 and P1(Ac-EESTSFQGP-CONH_2_)/P-1(Ac-EAQTRL-OH) in the two sequences show different interactions with surface residues on SHANK1; (**F**) magnified figure showing that Asp706^PDZ^ interacts with Gln (1) of Ac-EESTSFQGP-NH_2_ and Arg(-1) in GKAP PBM, respectively.

To obtain further insight into the molecular basis of the SHANK1/Ac-EESTSFQGP-NH_2_ interaction, the co-crystal structure was solved at a resolution of 1.98 Å (Supplementary Table S2). The crystals used for structure determination belonged to the trigonal space group *P*3_2_21 (Supplementary Figures S4 and S5 and Table S3). As the interactions of *C*-terminal PBMs with PDZ domains have been well studied, we selected the complex between *GKAP* PBM (Ac-EAQTRL-COOH) [8] and SHANK1 (PDB: 1Q3P) as a representative model for comparison with the internal ligand-mediated interaction. Due to the similar target-ligand binding modes within the four individual protomers of the SHANK1/Ac-EESTSFQGP-NH_2_ complex (Supplementary Table S4), chain A and chain E of protomer I were used to align with chain B and chain D from the SHANK1/*GKAP* PBM structure (Figure 3A) with the two ligands’ residues positioned -5 to 3 (P-5 to P3) from the *N*- to *C*- terminus. Overall, the two SHANK1 backbones exhibit extensive overlap with an RSMD of 0.45 Å, with subtle differences within the loops, e.g. Q667-G675^PDZ^, G680-Q700^PDZ^, V705-G709^PDZ^ and G748-T748^PDZ^. The overall divergence likely arises from the different *N*-terminal lengths of βA (RMSD of 0.55 Å), associated with different *N*-terminal lengths of the expressed PDZ proteins in the two models (Supplementary Table S5 for RMSD calculations associated with specific regions of the SHANK1 sequence). Loop flexibility likely contributes to adaptation for binding of the different ligands; comparing the SHANK1/Ac-EESTSFQGP-NH_2_ and SHANK1/*GKAP* PBM structures, the loops in the Ac-EESTSFQGP-NH_2_ bound PDZ appear slightly extended so as to accommodate the *N*-terminal acetyl group as well as the *C*-terminal extended motif [Gln (1) — Gly (2) — Pro (3)] of the internal PBM. Comparison with syntrophin PDZ/ligand complexes [12,21,22] which provided the first models for internal ligands [12] and Par-6 PDZ/ligand complexes [23,24] further support this hypothesis. The superimposed crystal structures of Syntrophin PDZ/ TRPV3 (C-terminal, PDB code: 7QQN) [21] and Syntrophin PDZ/nNOS (internal, PDB code: 1QAV) [12] (Figure 3B and Supplementary Figure S6A) and Syntrophin PDZ/C-terminal peptide (PDB code: 2PDZ) [22] and Syntrophin PDZ/nNOS (internal, PDB code: 1QAV) [12] (Supplementary Figure S6B), highlight that the protein backbones also overlap (RMSD of 0.79 and 1.15 Å), with divergence primarily in the loop from R85-I94 (RMSD of 2.37 and 1.83 Å, Supplementary Tables S6 and S7), which is in the vicinity of the key αB-βB pocket accommodating the ligands. We also superimposed the crystal structures of Par-6 PDZ/C-terminal peptide (PDB code: 1RZX) [21,24] and Syntrophin PDZ/pals1 (internal, PDB code: 1X8S) [23] (Supplementary Figure S6C) with a full-length RMSD of 0.54 Å. In line with the emphasis on conformational changes in the carboxylate loop due to the binding of ligands, the major divergence comes from the loop L164-F174 with RMSD of 2.03 Å (Supplementary Table S8). In addition, the previously reported structure of the SHANK1-PDZ/*ARAP3* fusion protein shows similar behaviour in terms of conformational changes in the PDZ domain [14], as does our previously reported structure of the SHANK1 PDZ domain bound to a GKAP Leu(P0)Phe variant [19]. Considering the ITC, VT-FA, and thermal melting results, the observation of extended loops in the structure described here may account for the less favourable entropic cost of binding Ac-EESFSTQGP-CONH_2_ to SHANK1 in comparison with *GKAP* PBM. It should be noted that the importance of allostery and protein flexibility has also recently been highlighted in an NMR study probing the molecular mechanism by which hPTP1E-PDZ2 recognizes RA-GEF2 [14].

Crucially, one of the most obvious features of *C*-terminal PBM recognition is the insertion of the *C-*terminal carboxylate group of the residue at P0 into the PDZ conserved carboxylate binding loop. Both Phe (0) of the internal ligand and Leu (0) of the *C*-terminal PBM form hydrogen bonds with three target carboxylate-binding-loop residues, F674^PDZ^, G675^PDZ^ and F676^PDZ^, thus contributing significantly to the low-micromolar SHANK1 affinities (Figure 3C,D). Both amide-Hs in Phe (0) and Leu (0) form a hydrogen bond with the carbonyl group of F676 ^PDZ^ (Figure 3D). The interaction between one of the oxygens on the carboxylate of Leu^0^ to the amide-Hs of G675^PDZ^ and the other to the amido-H of F674^PDZ^, differs from that which is observed for Phe (0) whereby the C=O interacts in a bifurcated manner with the amido-H of G675^PDZ^ and amido-H of F674^PDZ^. This suggests replacement of the *C*-terminal residue Leu (0) with the amidated Phe (0) only results in local atomistic differences and does not substantially interfere with the ligands’ interactions with the conserved hydrophobic pocket further confirming the *C*-terminal plasticity of SHANK1-PDZ binders. The residues at P-5 and P-2 of the two PBMs exhibit similar conformations and interact with identical target residues (Figure 3C). For example, Glu (-5) hydrogen-bonds to Tyr701^PDZ^ and Arg679^PDZ^ and Thr (-2) to Leu678^PDZ^ and His735^PDZ^. The remaining residues at P-4, P-3 and P-1 differ in their non-covalent interactions with SHANK1. As shown in Figure 3E, Ac-EESTSFQGP-NH_2_ Glu (-4) and GKAP PBM Ala (-4) interact with Gly680^PDZ^, whilst the former additionally interacts with Arg736^PDZ^, which may contribute to the more favourable enthalpic binding for Ac-EESTSFQGP-NH_2_ compared with GKAP PBM as observed in ITC experiments. At P-3, even though both residues participate in interactions with SHANK1, Ac-EESTSFQGP-NH_2_ Ser(-3) forms a hydrogen bond to Glu703^PDZ^ (2.6 Å) whereas GKAP PBM Gln (-3) interacts with Arg679^PDZ^ (3.1 Å) (Supplementary Table S4). Interestingly, Ser(-1) of Ac-EESTSFQGP-NH_2_ is not involved in interaction and instead Gln (1) compensates by binding to Asp706^PDZ^ — a role fulfilled by Arg(-1) in GKAP PBM (Figure 3F). Finally, Gly (2) and Pro (3) on the *C*-terminus of Ac-EESTSFQGP-NH_2_ do not appear to interact with any residues on the PDZ domain indicating these sites represent options for truncation or modifications of the Ac-EESTSFQGP-NH_2_ sequence in the future.

## Conclusion

In the present study, we selected a 9-mer linear motif EESTSFQGP as an internal PBM ligand and investigated its interaction with the human SHANK1-PDZ domain (656–762). EESTSFQGP was shown to bind to SHANK1 with low µM affinity; comparable potency to a representative *C*-terminal PBM (*GKAP*), and, compete for the same binding site as shown by direct and competition FA assays. ITC and VT-FA analyses revealed the internal ligand to bind with more favourable enthalpy and less favourable entropy in comparison with *GKAP*. X-ray crystallographic analyses on the SHANK1/Ac-EESTSFQGP-CONH_2_ complex identified an asymmetric tetramer with four similar protein-ligand protomers. Comparison between the Ac-EESTSFQGP-CONH_2_ and GKAP complexes reveals a similar conformation of the PDZ domain in both cases (RMSD of 0.45 Å), and that subtle differences in loop regions and local hydrogen-bonding interaction allow the amide of Phe (0) in Ac-EESTSFQGP-CONH_2_ to effectively substitute for the Leu (0) carboxylate of GKAP, offering a possible explanation for the less favourable entropy of binding of the former. Similarly, Ac-EESTSFQGP-CONH_2_ was shown to engage in additional non-covalent interactions with PDZ residues compared to *GKAP* PBM and may explain the higher enthalpy of interaction. Published studies on thermodynamic signatures for PDM/PDZ interactions consistently show enthalpy entropy-compensation, for instance where the length of PBM is varied [25] or the C-terminal residue of the PBM is varied [26], where a PDZ binds to class I, II or III type PBMs (incl. sequences with the capacity to interact through an internal mode) [27], or, where different PBMs bind to the same PDZ [28]. The results presented here emphasize the ability of the PDZ domain to readily adapt to different ligand classes. Finally, the residues Gly (2) and Pro (3) do not appear to interact with SHANK1 in Ac-EESTSFQGP-CONH_2_ suggesting the potential to further truncate this sequence and use internal ligands as alternative templates for design of SHANK1-PDZ modulators.

## Experimental procedures

### Peptide synthesis, purification and characterization

All peptides were prepared using a CEM Liberty Blue automated microwave synthesizer based on the Fmoc-based solid phase peptide synthesis method. Rink-Amide MBHA resin (100–200 mesh) was used with a loading of 0.35 mmol/g. All Fmoc protected amino acids and coupling reagents were purchased from Merck or from Fluorochem. The procedures included four major steps: swell, wash, deprotection and amino acid coupling. Dichloromethane-treated resin was deprotected using 20% pyridine in DMF so that the *N*-terminal amine group was exposed. The reagents DIC (5 equiv. in DMF) and Oxyma Pure (5 equiv. in DMF) were used to couple with the corresponding amino acids at 90°C for 5 min. The coupling step was repeated twice and the deprotection followed alternately. Following every step, the resin was washed using DMF three times and dried.

*N*-terminally acetylated peptides were obtained by treatment with acetic anhydride−DIPEA (1:1, v/v, 10 equiv. in DMF) at room temperature for 30 min, followed by two washes with DMF and DCM solvents. *N*-terminally fluorescently labelled peptides were obtained by elongating the *N*-terminus with Fmoc-Ahx-OH (5 equiv. in DMF) then 5(6)-Carboxyfluorescein or fluorescein isothiocyanate (FITC) (5 equiv. in DMF) respectively; both with DIC (9 equiv. in DMF) and HOBt (5 equiv. in DMF) as coupling reagents at room temperature for 4 h.

After synthesis, all peptides were obtained by treatment with cleavage cocktail (TFA/triisopropylsilane/2,2-(Ethylenedioxy)diethanethiol/water, 92.5:2.5:2.5:2.5, v/v/v/v) at room temperature for 3.5 h. Peptide products were precipitated using cold diethyl ether and then centrifuged. Precipitations were collected and dissolved in 10% acetonitrile in water then freeze-dried for purification. An Agilent 1260 infinity HPLC and Bruker maXis II™ ESI–QTOF mass spectrometer were used to purify the crude peptides and acquire analytical HPLC and high-resolution mass spectrometry data respectively. See Supporting Information 2.1, 2.2, 2.5 for characterization data.

### Protein overexpression and purification

Human SHANK1 PDZ domain (656–762) was prepared as described previously [12]. Briefly, the domain was cloned into the pGEX-6P-2 expression vector and transformed into BL21 Gold cell lines for expression. Ten millilitres of overnight starter culture was inoculated in 1 l commercially available LB broth (Miller) containing 50 μg/ml chloramphenicol. Cells were incubated with shaking at 37°C until OD_600_ 0.6–0.8 then induced with 0.1 mM IPTG overnight at 18°C. Cell pellets were harvested and resuspended in 20 mM Tris, pH 8, 500 mM NaCl buffer containing 1 mg lysozyme, 0.5 mg DNAse and 1/6 cOmplete™, mini EDTA-free protease inhibitor cocktail tablet. Cells were then lysed by sonication (8 cycles, 20 s on 40 s off, 10 μA) and centrifuged at 30 000 ***g*** RCF for 25 min at 4°C. The supernatant was filtered using a 0.45 μm membrane and applied to glutathione beads. Ten column volumes of 20 mM Tris, pH 8, 500 mM NaCl buffer were used to wash the beads then 20 column volumes of elution buffer of 20 mM Tris, pH 8, 150 mM NaCl, 25 mM glutathione. Collected fractions were analysed by SDS–PAGE. PreScission protease was added to the elution fraction and the GST tag was cleaved overnight at 4°C. The elution fraction was then concentrated and reapplied to glutathione beads. The new eluted fraction was purified by size-exclusion chromatography on S75 26/60 pg column in 20 mM Tris, 150 mM NaCl, pH 7.5 buffer. Pure protein was analysed by high resolution mass spectrometry: expected *m/z* = 12 326.3 measured *m/z* = 12 325.6. Concentration was determined by Nanodrop using 8480 M^−1 ^cm^−1^ as the extinction coefficient. Related data are shown in the Supporting Information 2.3 and 2.4.

### Fluorescence anisotropy

#### Direct titration

Direct titration assays were performed in 384-well plates (Greiner Bio-one). Two hundred micromolars of SHANK1 protein was dialyzed into the assay buffer before use. Twenty microlitres of the assay buffer (50 mM ammonium-acetate, pH 6.5) was first added to each well. Twenty microlitres of the target protein sample was then added to the first column, followed by a two-fold serial dilution over 24 points. Twenty microlitres of 50 nM tracer peptide or 20 µl of the assay buffer was added to the corresponding row wells. The titration was performed in triplicate. Plates were read immediately, and after an hour or after 24 h on a PerkinElmer EnVision™ 2103 MultiLabel plate reader, with excitation at 480 nm (30 nm bandwidth), polarized dichroic mirror at 505 nm and emission at 535 nm (40 nm bandwidth, S and P polarized) at a controlled temperature of 25°C. The P (perpendicular intensity) and S (parallel intensity) channels raw data were obtained. The data processing and formulae are shown in the Supporting Information 2.6.

#### Competition assays

FA competition assays were also performed in 50 mM NH_4_OAc, pH 6.5 buffer in 384-well plates. Twenty microlitres of the assay buffer was first added to wells. Twenty microlitres of 5000 µM competitor peptides was added to the first columns, followed by a twofold serial dilution over 16 points. Three micromolars of SHANK1 protein was dialyzed into the assay buffer before use. Twenty microlitres of the SHANK1 protein was added to each well to give a final protein concentration of 1 μM. Twenty microlitres of 50 nM tracer peptides or 20 µl of the assay buffer was added to corresponding row wells. The titration was performed in triplicate. Plates were read immediately with an excitation and emission wavelength of 480 and 535 nm respectively (dichroic mirror 505 nm), and after an hour or after 24 h on the plate reader with the same parameters as described above. The data processing and formulae are shown in the Supporting Information 2.7.

#### VT-FA assays

VT-FA assays were performed in 384-well plates (Greiner Bio-one). Six hundred micromolars of SHANK1 protein was dialyzed into different assay buffers before use. In this experiment, we prepared six buffers, including Buffer 1–25 mM HEPES [4-(2-hydroxyethyl)-1-piperazineethanesulfonic acid], 150 mM NaCl, pH 7.5, Buffer 2–25 mM phosphate, 150 mM NaCl, pH 7.5, Buffer 3–25 mM MOPS [3-(N-morpholino)propanesulfonic acid], 150 mM NaCl, pH 7.5, Buffer 4–25 mM HEPES, pH 7.5, Buffer 5–25 mM HEPES, 150 mM NaCl, pH 6.0, and Buffer 6–25 mM HEPES, 150 mM NaCl, pH 8.0. Twenty microlitres of the assay buffer was first added to each well. Twenty microlitres of the target protein sample was then added to the first column, followed by a twofold serial dilution over 24 points. Twenty microlitres of 50 nM tracer peptide or 20 µl of the assay buffer was added to the corresponding row wells. Plates were read at increasing intervals of 2.5°C, following a minimum period of 5 min equilibration at each temperature. The Δ*G*, Δ*H* and −T*Δ*S* were further calculated.

### Isothermal titration calorimetry (ITC)

A Microcal ITC200i instrument (Malvern) was employed to carry out the ITC experiments. Prior to use, SHANK1 protein was dialyzed against 20 mM Tris, 150 mM NaCl, pH 7.5 buffer. Lyophilized test peptides were dissolved in the same buffer to give a final concentration of 500 µM. Sixty microlitres of every test peptide was placed in the syringe to titrate against 350 µl of 50 µM Shank1 protein in the cell. The injection volumes were 2 µl each, injection time 6 s, and a 120 s delay between each injection for 20 injections in total. The titration for peptide into buffer was subtracted from the titration for peptide into protein to account for the heat of peptide dilution. Raw data was processed in the software Microcal Origin 8 and fit to a one-binding site model.

### Melting temperature determination (*T*_m_)

Samples of the complexes of SHANK1 PDZ protein and its ligands were prepared in 50 mM phosphate buffer pH 7.5 at 50 µM concentration. Circular dichroism spectra were acquired from 190 to 260 nm (step size of 1 nm, bandwidth 2 nm) from 20°C to 90°C in increments of 1°C at a heating rate of 1°C/min in a 1 mm path length quartz cuvette. The samples were measured three times and averaged. Data were converted to mean residue ellipticity and processed by subtracting buffer baseline spectrum. Multi-wavelength melt curves were collected on Chirascan Plus (Applied Photophysics) spectropolarimeter and processed using Global 3 Thermal Global Analysis Software.

### Crystallization

SHANK1 protein and its ligands were mixed in a 1:1.5 molar ratio in 25 mM Tris, 150 mM NaCl, pH 7.5 buffer, where the final protein concentration was 5 mg/ml. The crystals grew using the sitting drop, vapour diffusion method with crystallisation condition A10 of Morpheus screen (Molecular Dimensions), 20% v/v ethylene glycol, 10% w/v PEG 8000, 0.018 M magnesium chloride, 0.018 M calcium chloride, 0.1 M Tris pH 7.5, 0.1 M bicine pH 7.5. The crystallization solution was mixed with sample solution (1:1, v/v) with final drop volume of 0.2 μl. Crystals grew within 2–3 weeks at 20°C, and these were flash cooled in liquid nitrogen prior to data collection. Diffraction data were recorded on beamline i24 (wavelength 1.00 Å) at the Diamond Light Source at 100 K. The data for SHANK1 protein/Ac-EESTSFQGP-NH_2_ was processed and scaled using XIA2 [29] and DIALS [29]. The unit cell parameters for the crystal are *a* = *b* = 149.1 Å, *c* = 64.1 Å, α = β = 90°, γ = 120° in space group *P*3_2_21 with four SHANK1/Ac-EESTSFQGP-NH_2_ protomers in the asymmetric unit cell. The structure was determined by molecular replacement using Phaser [30] with the human Shank structure (PDB 6YWZ) monomer as the search model. Iterative cycles of manual model building using both 2*F_o_*-*F_c_* and *F_o_*-*F_c_* maps and refinement were carried out using COOT [31] and REFMAC [32], respectively. Structural validations were carried out using MolProbity [33]. The SHANK1/Ac-EESTSFQGP-NH_2_ structure has been deposited with the PDB code 8S1R. Data collection and refinement statistics are reported in Supplementary Table S2.

## Data Availability

All relevant data are included in the supporting information or available via the protein data bank accession number PDB ID: 8S1R.

## Open Access

Open access for this article was enabled by the participation of University of Birmingham in an all-inclusive *Read & Publish* agreement with Portland Press and the Biochemical Society under a transformative agreement with JISC.

## Abbreviations

FA: fluorescent anisotropy
PBM: PDZ binding motif
PPI: protein–protein interaction
PSSM: position-specific scoring matrice
SLiM: short linear motif
VT: variable temperature
