# Supplementary material for: Biophysical and structural analyses of the interaction between the SHANK1 PDZ domain and an internal SLiM
Source: Biochem J. 2024 Jul 10;481(14):945–55. doi: 10.1042/BCJ20240126 (PMC11346428; doi:10.1042/BCJ20240126)
Supplement: Supplementary Material [file BCJ-481-945-s1.pdf]

Supporting Information

Biophysical and Structural Analyses of the Interaction between the SHANK1 PDZ Domain and an Internal SLiM

Yue Li[1,2], Chi H. Trinh,[2,3] Amanda Acevedo-Jake,[4] Diana Gimenez,[4] Stuart L. Warriner[1,2], Andrew J. Wilson[1,2,4]

[1] School of Chemistry, University of Leeds, Woodhouse Lane, Leeds LS2 9JT, UK

[2] Astbury Centre for Structural Molecular Biology, University of Leeds, Woodhouse Lane, Leeds LS2 9JT, UK

[3] School of Molecular and Cellular Biology, University of Leeds, Woodhouse Lane, Leeds LS2 9JT, UK

[4] School of Chemistry, University of Birmingham, Edgbaston, Birmingham B15 2TT, UK

Correspondence: Andrew J. Wilson, School of Chemistry, University of Birmingham, Edgbaston, Birmingham B15 2TT, UK. Email: a.j.wilson.1@bham.ac.uk

Table of Contents

1. Supplementary tables and figures 5

Table S1. Selection of the tested internal ligands. 5

Figure S1. Fluorescence anisotropy direct titration of SHANK1-PDZ in 50 mM NH_4OAc, pH 6.5 buffer using 50 nM FAM-Ahx-ARAP3$_{1414-1429}$-$CONH_2$ (12.2 ± 8.3 µM), FAM-Ahx-ELFN1$_{579-594}$-$CONH_2$ (3.6 ± 0.7 µM), and FAM-Ahx-EESTSFQGP-$CONH_2$ (0.8 ± 0.1 µM) as tracers. Plates was read after 24 hr incubation at room temperature. 5

Figure S2. VT-FA assay results of the SHANK1-PDZ with its *N*-terminally fluorophore-labelled internal ligand FAM-Ahx-EESTSFQGP-$CONH_2$ and *C*-terminal ligand FITC-Ahx-EAQTRL-OH. The K_d values were collected from the results of titrating 600 µM SHANK1-PDZ to 50 nM tracers in different buffers (Buffer 1: 25 mM HEPES [4-(2-hydroxyethyl)-1-piperazineethanesulfonic acid], 150 mM NaCl, pH 7.5; Buffer 2: 25 mM phosphate, 150 mM NaCl, pH 7.5; Buffer 3: 25 mM MOPS [3-(N-morpholino)propanesulfonic acid], 150 mM NaCl, pH 7.5; Buffer 4: 25 mM HEPES, pH 7.5; Buffer 5: 25 mM HEPES, 150 mM NaCl, pH 6.0; Buffer 6: 25 mM HEPES, 150 mM NaCl, pH 8.0) at 25 °C, 27.5 °C, 30 °C, 32.5 °C, 35 °C, 37.5 °C and 40 °C. The ΔG, ΔH and -T*ΔS were further calculated using the Arrhenius equation and the Gibbs free energy change equation. 6

Figure S3. Normalized-fitted temperature melting curves of apo SHANK1-PDZ, SHANK1-PDZ/Internal ligand Ac-EESTSFQGP-$CONH_2$ complex and SHANK1-PDZ/C-terminal ligand Ac-EAQTRL-OH complex. 7

Table S2. Collection and refinement statistics for obtained SHANK1-PDZ/Ac-EESTSFQGP-$CONH_2$ co-crystal structure 8

Table S3. Observed residual interactions between protomers. 9

Figure S4. Observed complex of four SHANK1/Ac-EESTSFQGP-NH_2 protomers I, II, III and IV stacking in one asymmetric unit cell. 10

Figure S5. Observed interactions between different protomers: **a.** Antiparallel interaction of β-strands between protomer I and II occurs from residues Leu653^{PDZ}, Ser655^{PDZ}, Tyr657^{PDZ}, Ile659^{PDZ}, Glu661^{PDZ} and Thr663^{PDZ} from Chain A and Chain B (distances of 2.9 – 3.0 Å); **b.** Protomer I interacts with protomer III by forming one hydrogen bond (3.1 Å) between Chain

A Glu707^{PDZ} and Chain G Ser(-1); **c.** Protomer III contacts protomer IV by forming four pairs of hydrogen bonds between Chain C residues Arg713^{PDZ}, Gln667^{PDZ}, Met750^{PDZ}, Leu665^{PDZ} and Chain D residues Gln667^{PDZ}, Ser671^{PDZ}, Glu707^{PDZ}, Gly708^{PDZ}. 10

Table S4. Observed residual interactions between the internal PBM Ac-EESTSFQGP-CONH$_2$ and SHANK1 PDZ domain. 11

Table S5. RMSD values calculated for different parts of the sequences for the SHANK1/GKAP PBM (grey, PDB code: 1Q3P) and SHANK1/Ac-EESTSFQGP-CONH$_2$ (green, PDB code: 8S1R) structures. 13

Figure S6. Superimposed crystal structures of (a) Syntrophin PDZ (light grey)/C-terminal PBM (light orange, PDB code: 7QQN) and Syntrophin PDZ (light green)/Internal ligand (light cyan, PDB code: 1QAV) with a full-length calculated RMSD of 0.79 Å; (b) Syntrophin PDZ (light grey)/C-terminal PBM (yellow, PDB code:2PDZ) and Syntrophin PDZ (light green)/Internal ligand (light cyan, PDB code: 1QAV) with a full-length calculated RMSD of 1.153 Å and (c) Par-6 PDZ (dark grey)/C-terminal PBM (salmon orange, PDB code:1X8S) and Par-6 PDZ (smudge green)/Internal ligand (teal cyan, PDB code: 1RZX) with a full-length calculated RMSD of 0.542 Å. 14

Table S6. RMSD values calculated for different parts of the sequences for the Syntrophin PDZ/C-terminal PBM (PDB code: 7QQN) and Syntrophin PDZ/Internal terminal PBM (PDB code: 1QAV) structures. 15

Table S7. RMSD values calculated for different parts of the sequences for the Syntrophin PDZ/C-terminal PBM (PDB code: 2PDZ) and Syntrophin PDZ/Internal terminal PBM (PDB code: 1QAV) structures. 16

Table S8. RMSD values calculated for different parts of the sequences for the Par-6 PDZ/C-terminal PBM (PDB code: 1X8S) and Syntrophin PDZ/Internal terminal PBM (PDB code: 1RZX) structures. 17

2. Characterisation data 18

2.1 Mass spectra data of the tested peptides 18

2.2 HPLC analytical purity data of the tested peptides 24

2.3 SDS-PAGE gel for the SHANK1 PDZ protein expression and purification 27

2.4 Mass spectrum of the SHANK1 PDZ protein expression 27

2.5 Chromatographic conditions for preparative HPLC$^{\alpha}$ 27

$^{\alpha}$. Reversed Phase C18 column, mobile phase A (0.1% TFA in water), mobile phase B (0.1% TFA in ACN). 28

2.6 Equations for data calculation in fluorescent anisotropy direct titration 28

2.7 Equations for data calculation in fluorescent anisotropy competition assays 28

1. Supplementary tables and figures

Table S1. Selection of the tested internal ligands.

Name	Sequence
FAM-Ahx-ARAP3$_{1414-1429}$-CONH$_2$	FAM-Ahx-FPELIQDTSTSFSTTR-CONH$_2$
FAM-Ahx-ELFN1$_{579-594}$-CONH$_2$	FAM-Ahx-ESTSFQGVKSGPVSVA-CONH$_2$
Fluorophore-labelled SLiM	FAM-Ahx-EESTSFQGP-CONH$_2$
SLiM modelled by PSSM	Ac-EESTSFQGP-CONH$_2$

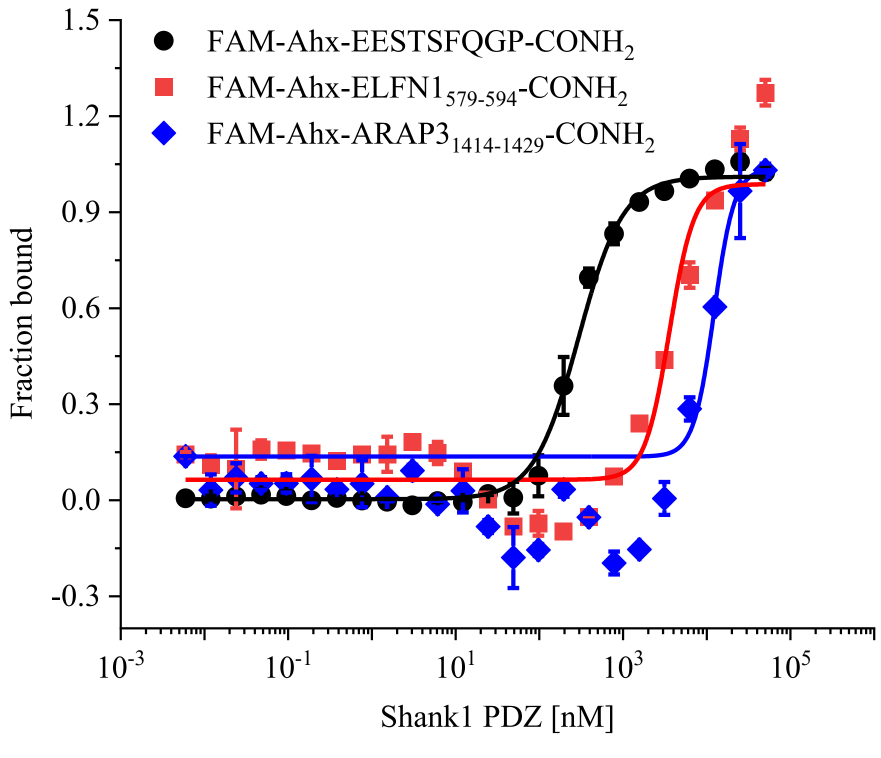

Figure S1. Fluorescence anisotropy direct titration of SHANK1-PDZ in 50 mM NH_4OAc, pH 6.5 buffer using 50 nM FAM-Ahx-ARAP3$_{1414-1429}$-CONH$_2$ (12.2 ± 8.3 μM), FAM-Ahx-ELFN1$_{579-594}$-CONH$_2$ (3.6 ± 0.7 μM), and FAM-Ahx-EESTSFQGP-CONH$_2$ (0.8 ± 0.1 μM) as tracers. Plates was read after 24 hr incubation at room temperature.

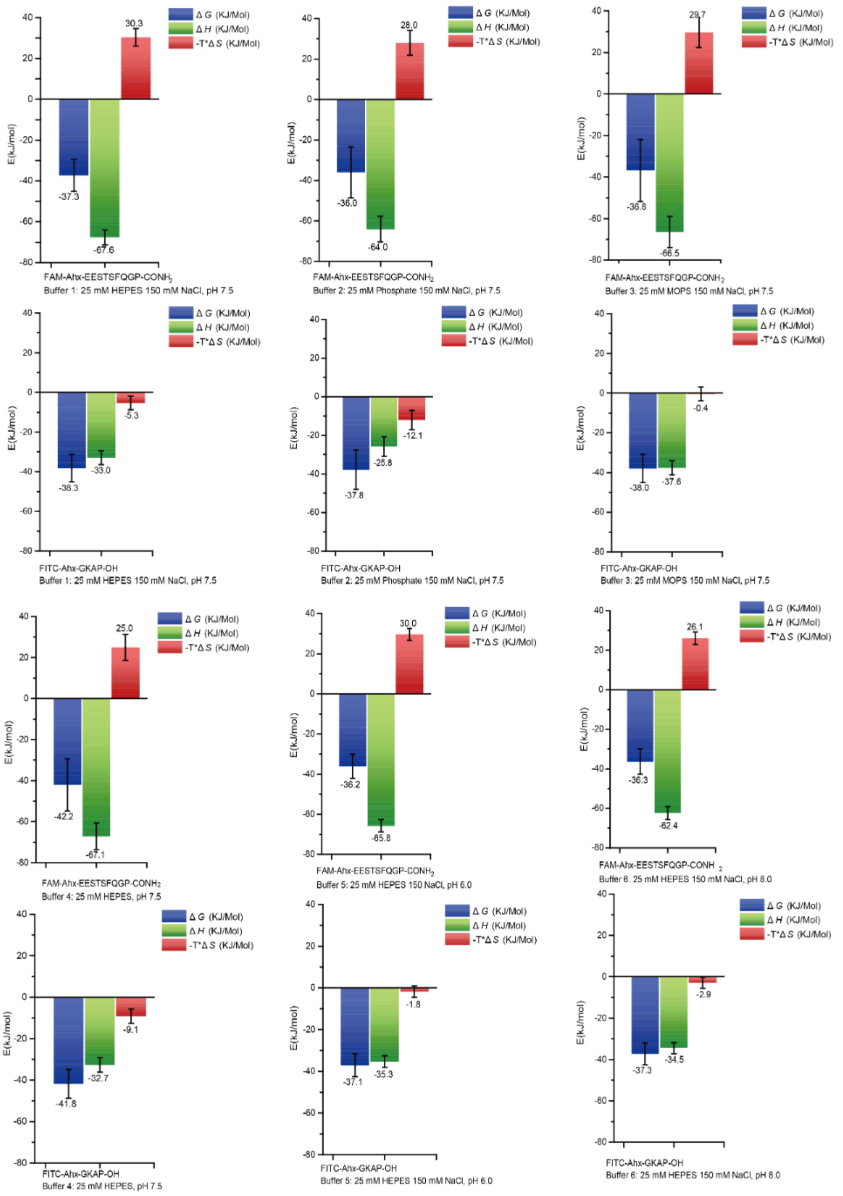

Figure S2. VT-FA assay results of the SHANK1-PDZ with its *N*-terminally fluorophore-labelled internal ligand FAM-Ahx-EESTSFQGP-$CONH_2$ and *C*-terminal ligand FITC-Ahx-EAQTRL-OH. The K_d values were collected from the results of titrating 600 μM SHANK1-PDZ to 50 nM tracers in different buffers (Buffer 1: 25 mM HEPES [4-(2-hydroxyethyl)-1-piperazineethanesulfonic acid], 150 mM NaCl, pH 7.5; Buffer 2: 25 mM phosphate, 150 mM NaCl, pH 7.5; Buffer 3: 25 mM MOPS [3-(N-morpholino)propanesulfonic acid], 150 mM NaCl, pH 7.5; Buffer 4: 25 mM HEPES, pH 7.5; Buffer 5: 25 mM HEPES, 150 mM NaCl, pH 6.0; Buffer 6: 25 mM HEPES, 150 mM NaCl, pH 8.0) at 25 °C, 27.5 °C, 30 °C, 32.5 °C, 35 °C, 37.5 °C and 40 °C. The Δ*G*, Δ*H* and -T*Δ*S* were further calculated using the Arrhenius equation and the Gibbs free energy change equation.

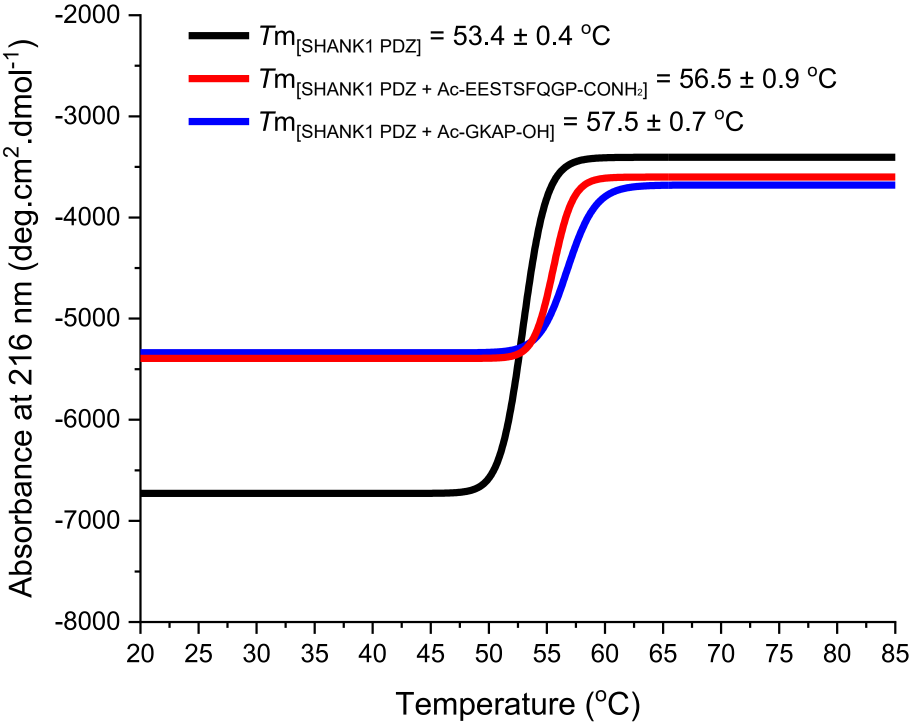

Figure S3. Normalized-fitted temperature melting curves of apo SHANK1-PDZ, SHANK1-PDZ/Internal ligand Ac-EESTSFQGP-$CONH_2$ complex and SHANK1-PDZ/C-terminal ligand Ac-EAQTRL-OH complex.

Table S2. Collection and refinement statistics for obtained SHANK1-PDZ/Ac-EESTSFQGP-CONH$_2$ co-crystal structure

Data Collection Statistics			
X ray source	DLS Beamline i24		
Average unit cell	a, b, c, (Å): 149.10, 149.10, 64.07<br>α, β, γ (°): 90, 90, 120		
Processed using	Xia2 dials		
/Space group	*P* 3 2 2 1		
	Overall	Inner shell	Outer shell
Low resolution limit	129.16	129.43	2.01
High resolution limit	1.98	5.37	1.98
R_{merge} (all I+ and I-)	0.123	0.061	1.661
R_{meas} (all I+ and I-)	0.129	0.064	1.754
R_{pim} (all I+ and I-)	0.039	0.019	0.560
Total number of obs	1184571	60633	54186
Total number unique	57199	3009	2827
Mean((I)/sd(I))	14.7	51.4	1.2
Completeness	100.0	100.0	100.0
Multiplicity	20.7	20.2	19.2
CC Half	0.999	0.999	0.831
Wilson B Factor (Å^2)	34.9		
Model Refinement			
Protein molecules in au	4		
Rwork/Rfree (%)	21.7/23.6		
**Number of atoms**			
Protein	3321		
Ligand	291		
Water	115		
**Mean B factors (Å)**			
Protein	50.7		
Ligand	49.2		
Water	42.5		
**R.m.s. deviations**			
Bond length (Å)	0.01		
Bond angles	1.79		
**Ramahandran statistics**			
% favoured	98.2		
% allowed	100.0		
% outliers	0		
PDB accession code	8S1R		

Table S3. Observed residual interactions between protomers.

**Interactions of protomer I Chain A: promoter II Chain B**	**Distance (Å)**
Thr663 main chain NH: Leu653 main chain C=O	3.0
Glu661 main chain NH: Ser655 main chain C=O	2.9
Ile659 main chain NH: Tyr657 main chain C=O	2.9
Ile659 main chain C=O: Tyr657 main chain NH	2.8
Tyr657 main chain C=O: Ile659 main chain NH	3.0
Tyr657 main chain NH: Ile659 main chain C=O	2.9
Ser655 main chain C=O: Glu661 main chain NH	3.0
Leu653 main chain C=O: Thr663 main chain NH	2.9
**Interactions of protomer I Chain A: promoter III Chain G**	**Distance (Å)**
Glu707 main chain OH: Ser^{-1} side chain OH	3.1
**Interactions of protomer III Chain C: promoter IV Chain D**	**Distance (Å)**
Arg713 side chain NH: Gln667 main chain C=O	3.2
Gln667 main chain C=O: Ser671 side chain NH	2.9
Met750 side chain CH: Glu707 side chain C=O	3.3
Leu665 main chain NH: Gly708 main chain C=O	2.9

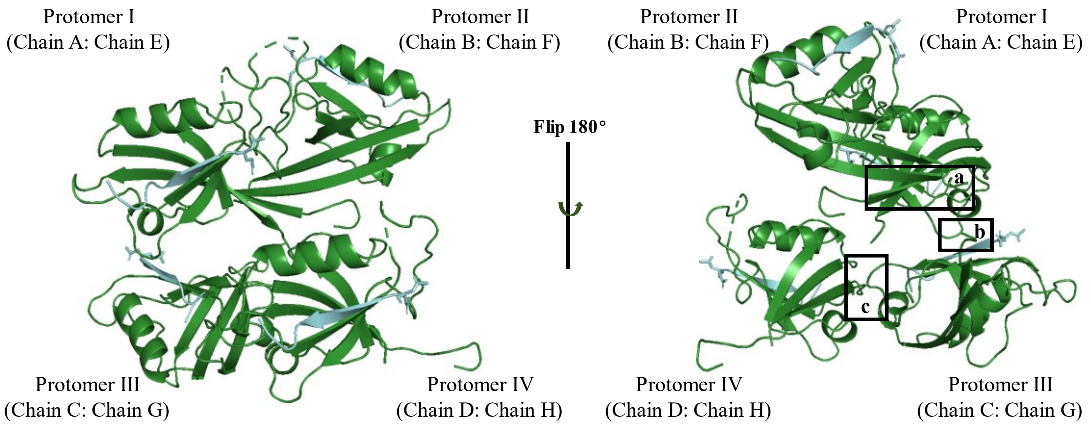

Figure S4. Observed complex of four SHANK1/Ac-EESTSFQGP-NH_2 protomers I, II, III and IV stacking in one asymmetric unit cell.

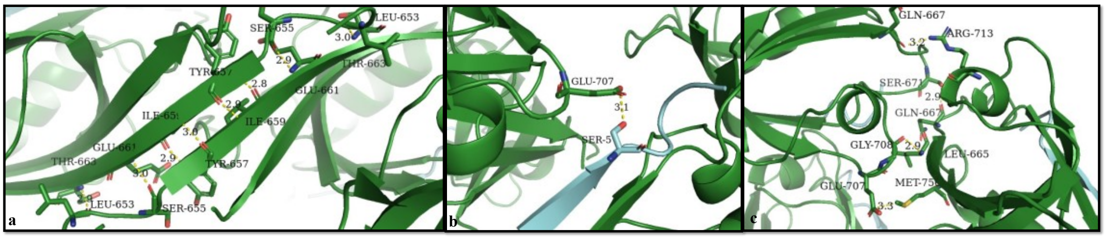

Figure S5. Observed interactions between different protomers: **a.** Antiparallel interaction of β-strands between protomer I and II occurs from residues Leu653^{PDZ}, Ser655^{PDZ}, Tyr657^{PDZ}, Ile659^{PDZ}, Glu661^{PDZ} and Thr663^{PDZ} from Chain A and Chain B (distances of 2.9 – 3.0 Å); **b.** Protomer I interacts with protomer III by forming one hydrogen bond (3.1 Å) between Chain A Glu707^{PDZ} and Chain G Ser(-1); **c.** Protomer III contacts protomer IV by forming four pairs of hydrogen bonds between Chain C residues Arg713^{PDZ}, Gln667^{PDZ}, Met750^{PDZ}, Leu665^{PDZ} and Chain D residues Gln667^{PDZ}, Ser671^{PDZ}, Glu707^{PDZ}, Gly708^{PDZ}.

Table S4. Observed residual interactions between the internal PBM Ac-EESTSFQGP-$CONH_2$ and SHANK1 PDZ domain.

**Interactions**	**Distance (Å)**			
Ligand-Protein	E-A	F-B	G-C	H-D
Glu^{-5}-$Tyr701^{PDZ}$ (Side chain OH to side chain OH)	2.9	3.0	3.3	2.9
Glu^{-5}-$Arg679^{PDZ}$ (Side chain C=O to side chain NH)	3.0	-	3.2	-
Glu^{-5}-$Arg679^{PDZ}$ (Side chain C=O to side chain NH_2)	3.1	-	-	-
Glu^{-4}-$Gly680^{PDZ}$ (Main chain NH to main chain C=O)	2.9	3.0	3.1	3.3
Glu^{-4}-$Gly680^{PDZ}$ (Main chain C=O to main chain NH)	3.0	3.1	3.1	3.0
Glu^{-4}-$Arg736^{PDZ}$ (Side chain C=O to side chain NH_2)	3.2	3.4	-	-
Glu^{-4}-$Arg736^{PDZ}$ (Side chain OH to side chain NH_2)	3.4	2.8	-	-
Glu^{-4}-$Arg736^{PDZ}$ (Side chain C=O to side chain NH)	-	3.2	2.6	-
Glu^{-4}-$Arg736^{PDZ}$ (Side chain OH to side chain NH)	-	3.3	3.0	-

Ser^{-3}-$Glu703^{PDZ}$ (Side chain OH to side chain C=O)	2.6	2.9	2.6	3.2
Thr^{-2}-$Leu678^{PDZ}$ (Main chain NH to main chain C=O)	2.8	2.8	2.8	3.4
Thr^{-2}-$Leu678^{PDZ}$ (Main chain C=O to main chain NH)	2.9	2.8	3.0	2.9
Thr^{-2}-$His735^{PDZ}$ (Side chain OH to imidazole ring H)	2.5	2.6	2.5	2.8
Ser^{-1}-$Glu707^{PDZ}$ (Side chain OH to main chain OH)	-			
Phe^{0}- $Phe674^{PDZ}$ (Main chain C=O to main chain NH)	3.1	3.0	3.1	3.0
Phe^{0}-$Gly675^{PDZ}$ (Main chain C=O to main chain NH)	2.9	2.8	2.9	3.0
Phe^{0}-$Phe676^{PDZ}$ (Main chain NH to main chain C=O)	2.7	2.7	2.9	2.8
Gln^{1}-$Asp706^{PDZ}$ (Side chain NH to side chain C=O)	3.0	2.9	2.8	3.4

Table S5. RMSD values calculated for different parts of the sequences for the SHANK1/GKAP PBM (grey, PDB code: 1Q3P) and SHANK1/Ac-EESTSFQGP-$CONH_2$ (green, PDB code: 8S1R) structures.

**Superimposed sequence**	**RMSD (Å)**
Full length	0.45
αA	0.097
αB	0.135
βA	0.552
βB	0.110
βC	0.125
βD	0.088
βE	0.029
βF	0.292
Loop V705-G709	0.342
Loop Q667-G675	0.311
Loop G745-T748	0.250
Loop N726-G727	0.025
Loop G680-Q700	0.629
Loop N729-G734	0.094
Loop A714-G719	0.150

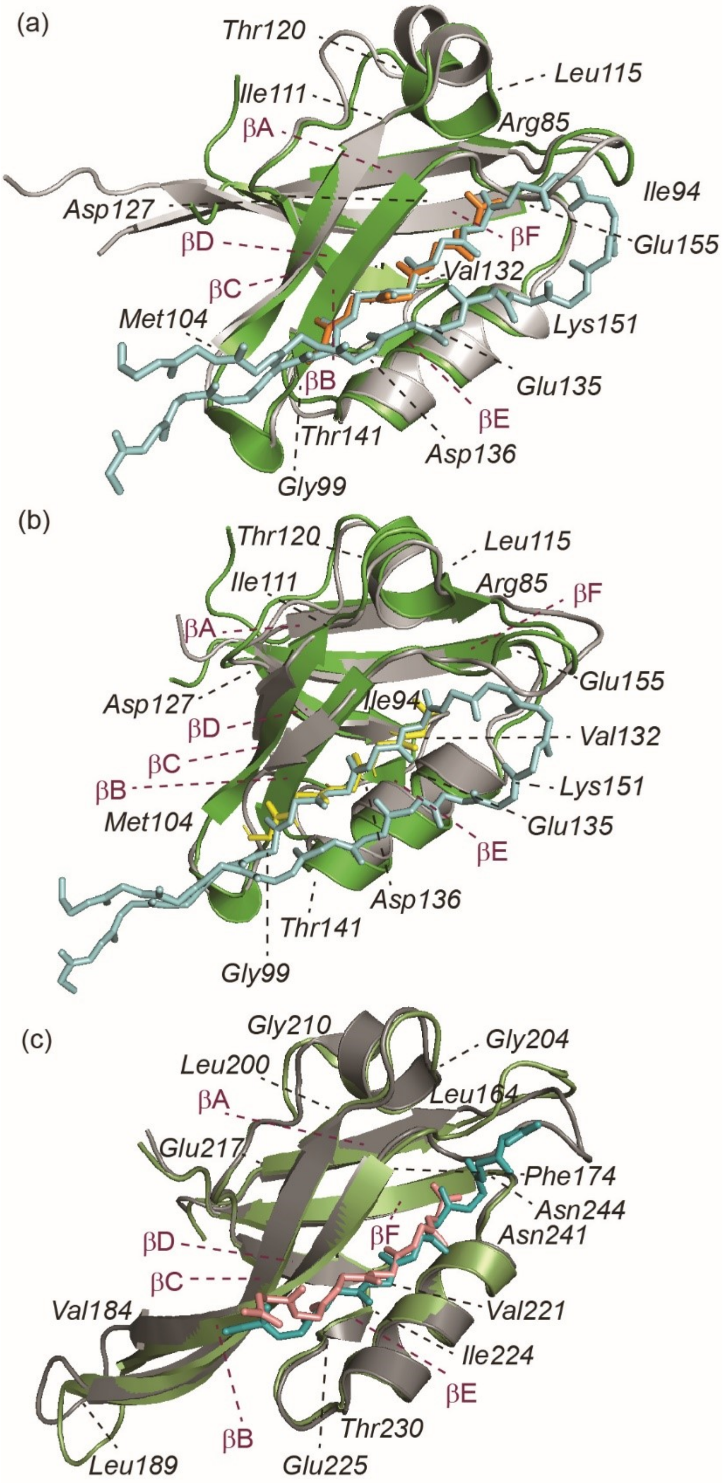

Figure S6. Superimposed crystal structures of (a) Syntrophin PDZ (light grey)/C-terminal PBM (light orange, PDB code: 7QQN) and Syntrophin PDZ (light green)/Internal ligand (light cyan, PDB code: 1QAV) with a full-length calculated RMSD of 0.79 Å; (b) Syntrophin PDZ (light grey)/C-terminal PBM (yellow, PDB code:2PDZ) and Syntrophin PDZ (light green)/Internal ligand (light cyan, PDB code: 1QAV) with a full-length calculated RMSD of 1.153 Å and (c) Par-6 PDZ (dark grey)/C-terminal PBM (salmon orange, PDB code:1X8S) and Par-6 PDZ (smudge green)/Internal ligand (teal cyan, PDB code: 1RZX) with a full-length calculated RMSD of 0.542 Å.

Table S6. RMSD values calculated for different parts of the sequences for the Syntrophin PDZ/C-terminal PBM (PDB code: 7QQN) and Syntrophin PDZ/Internal terminal PBM (PDB code: 1QAV) structures.

**Superimposed sequence**	**RMSD (Å)**
Full length	0.786
αA	0.160
αB	0.296
βA	0.163
βB	0.337
βC	0.474
βD	0.160
βE	0.000
βF	0.306
Loop R85-I94	2.365
Loop K151-E155	0.489
Loop V132-E135	0.041
Loop D136-T141	0.002
Loop G99-M104 (including αC)	0.157
Loop I111-L115	0.072
Loop T120-D127	0.183

Table S7. RMSD values calculated for different parts of the sequences for the Syntrophin PDZ/C-terminal PBM (PDB code: 2PDZ) and Syntrophin PDZ/Internal terminal PBM (PDB code: 1QAV) structures.

**Superimposed sequence**	**RMSD (Å)**
Full length	1.153
αA	0.156
αB	0.362
βA	0.410
βB	0.352
βC	0.601
βD	0.498
βE	0.016
βF	0.421
Loop R85-I94	1.829
Loop K151-E155	0.378
Loop V132-E135	0.054
Loop D136-T141	0.585
Loop G99-M104 (including αC)	0.314
Loop I111-F112	0.427
Loop T120-D127	0.768

Table S8. RMSD values calculated for different parts of the sequences for the Par-6 PDZ/C-terminal PBM (PDB code: 1X8S) and Syntrophin PDZ/Internal terminal PBM (PDB code: 1RZX) structures.

**Superimposed sequence**	**RMSD (Å)**
Full length	0.542
αA	0.271
αB	0.281
βA	0.478
βB	0.505
βC	0.443
βD	0.082
βE	0.001
βF	0.210
Loop L164-F174	2.032
Loop N241-N244	0.129
Loop V221-I224	0.072
Loop E225-T230	0.098
Loop V184-L189	0.378
Loop L200-G204	0.291
Loop G210-E217	0.190

2. Characterisation data

2.1 Mass spectra data of the tested peptides

FAM-Ahx-ELFN1$_{579\text{-}594}$

Exact Mass: 2048.9367

**Expected [M+2H$^+$]$^{2+}$**	**Measured [M+2H$^+$]$^{2+}$**
**1025.4764**	**1025.4750**
**Expected [M+3H$^+$]$^{3+}$**	**Measured [M+3H$^+$]$^{3+}$**
**683.9869**	**683.9843**

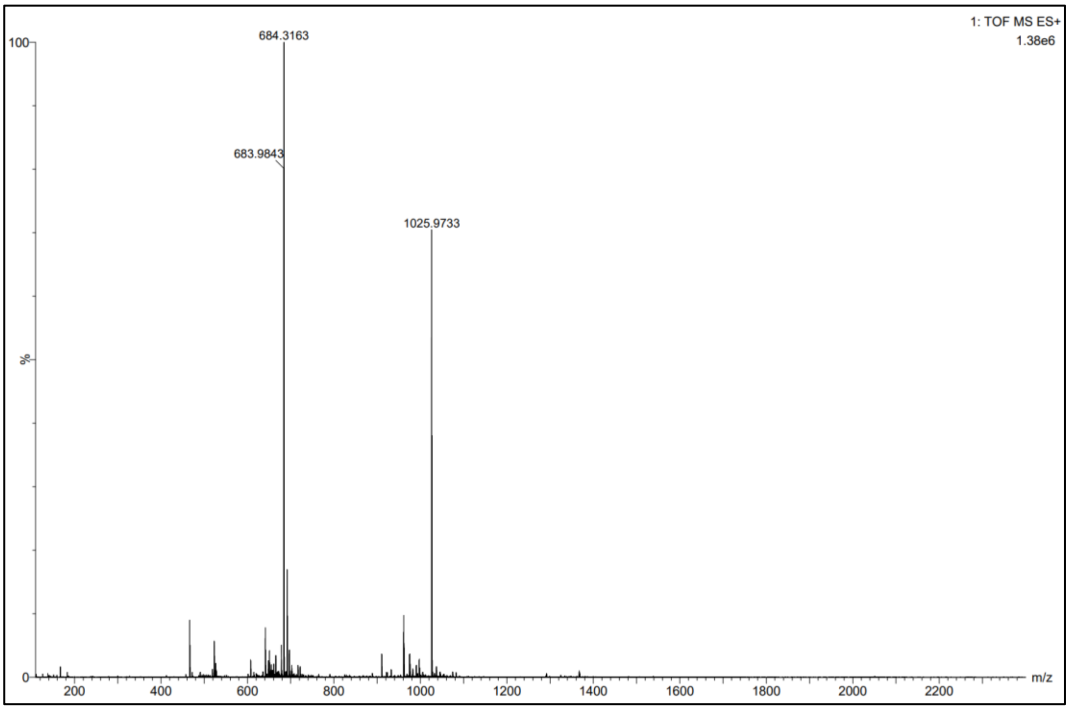

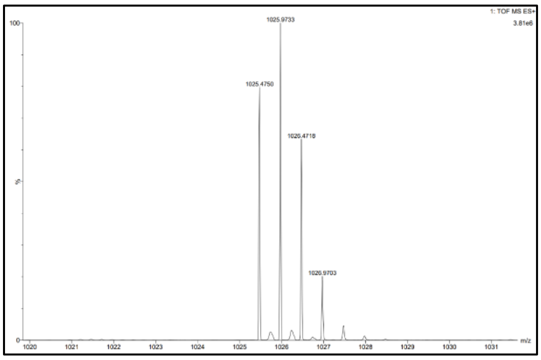

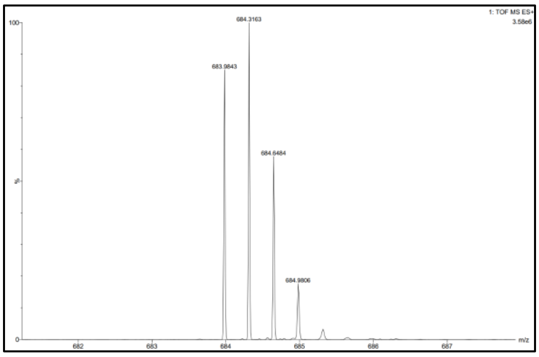

FITC-Ahx-GKAP-OH

Exact Mass: 1218.5016

Expected [M+2H$^+$]$^{2+}$	Measured [M+2H$^+$]$^{2+}$
610.2588	610.2538

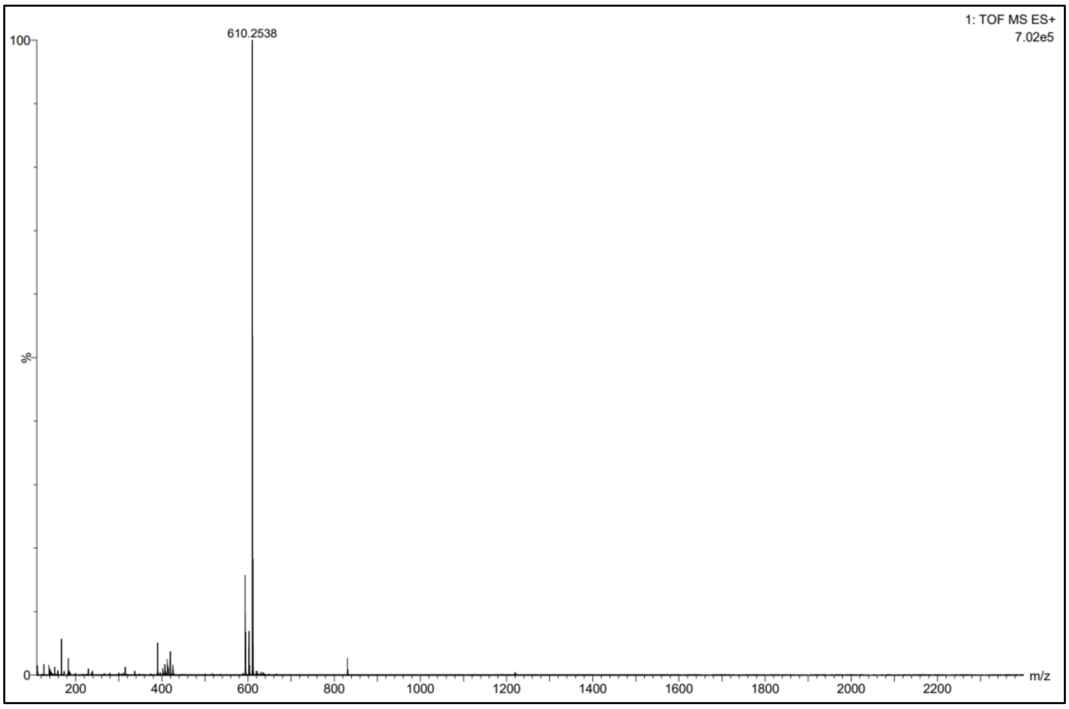

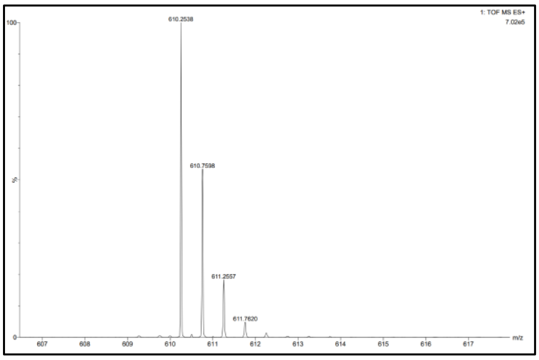

FAM-Ahx-ARAP3$_{1414\text{-}1429}$

Exact Mass: 2299.0321

Expected $[M+2H^+]^{2+}$	Measured $[M+2H^+]^{2+}$
1150.5241	1150.5227
Expected $[M+3H^+]^{3+}$	Measured $[M+3H^+]^{3+}$
767.3520	767.3421

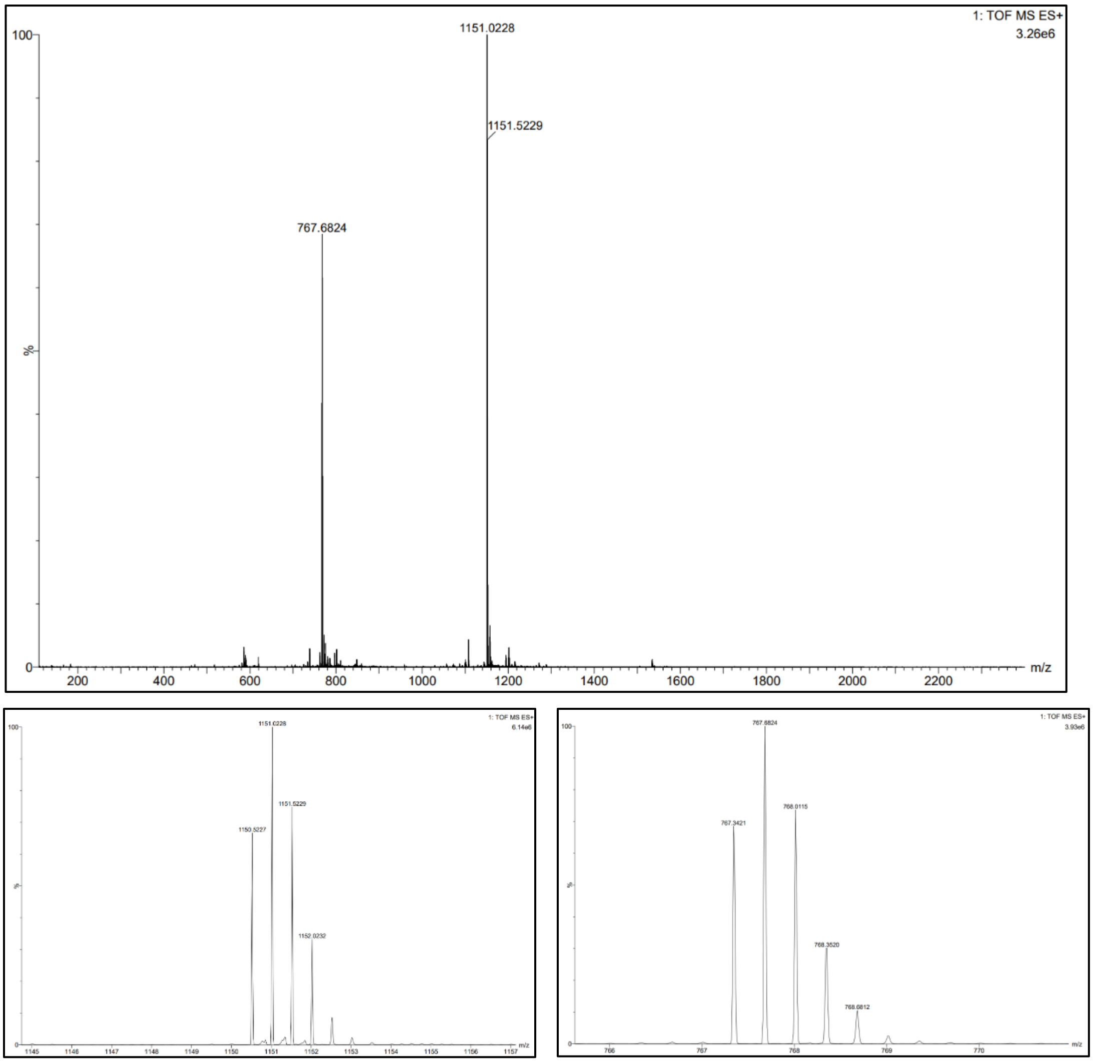

FAM-Ahx-EESTSFQGFP-CONH$_2$

Exact Mass: 1450.5565

**Expected $[M+2H^+]^{2+}$**	**Measured $[M+2H^+]^{2+}$**
**726.2783**	**726.2666**
**Expected $[M+H^++Na^+]^{2+}$**	**Measured $[M+H^++Na^+]^{2+}$**
**737.2668**	**737.2548**
**Expected $[M+2H^++K^+]^{3+}$**	**Measured $[M+2H^++K^+]^{2+}$**
**497.1784**	**497.1739**

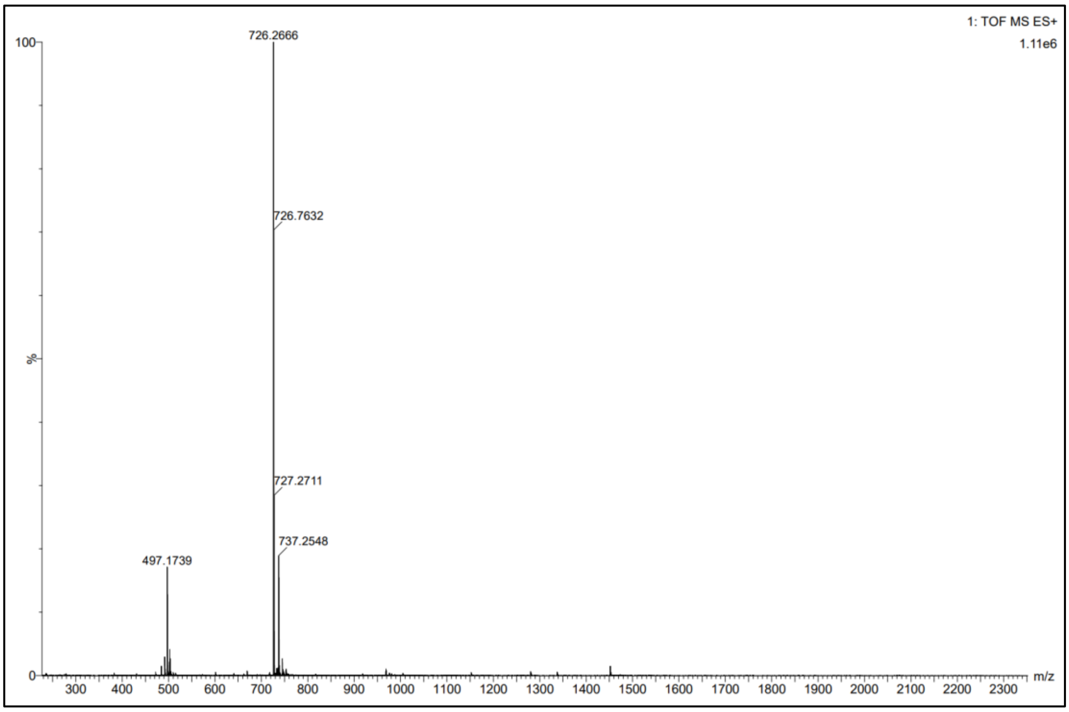

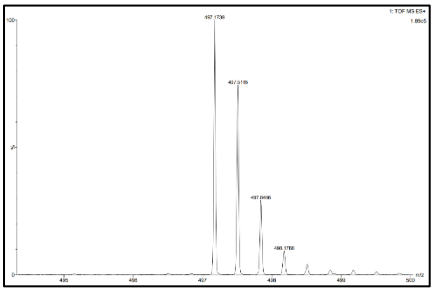

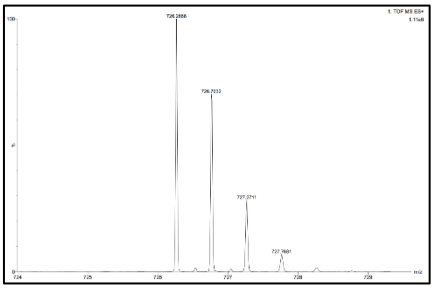

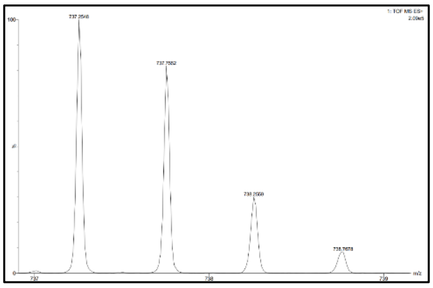

Ac-EESTSFQGP-CONH$_2$

Exact Mass:1021.4353

**Expected [M+H$^+$]$^+$**	**Measured [M+H$^+$]$^+$**
**1022.4425**	**1022.4435**
**Expected [M+2H$^+$]$^{2+}$**	**Measured [M+2H$^+$]$^{2+}$**
**511.7257**	**511.7238**

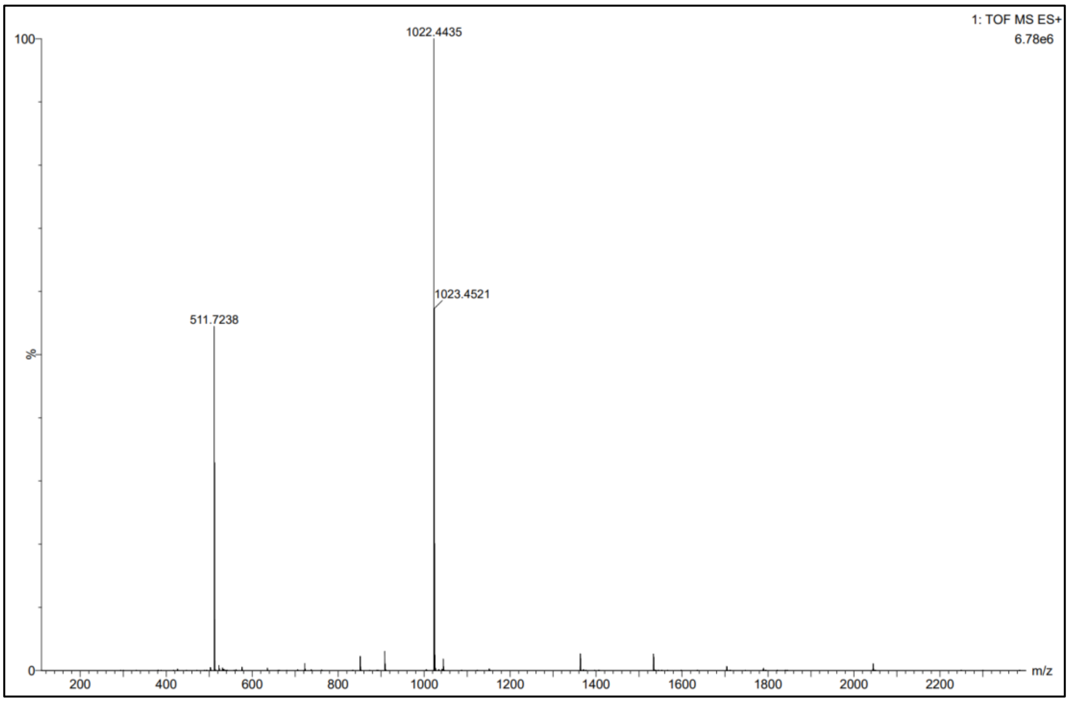

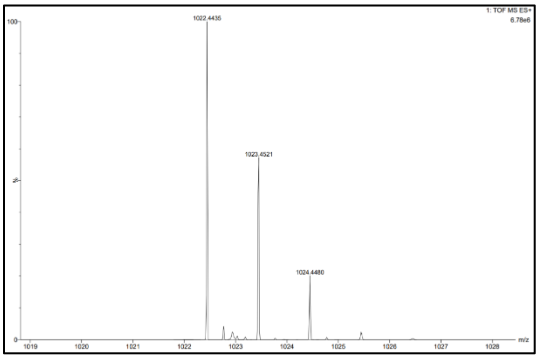

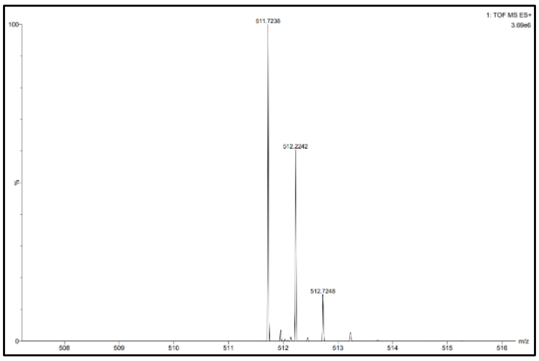

Ac-EAQTRL-CONH$_2$

Exact Mass:758.3923

Expected [M+H$^+$]$^+$	Measured [M+H$^+$]$^+$
**759.3995**	**759.4006**

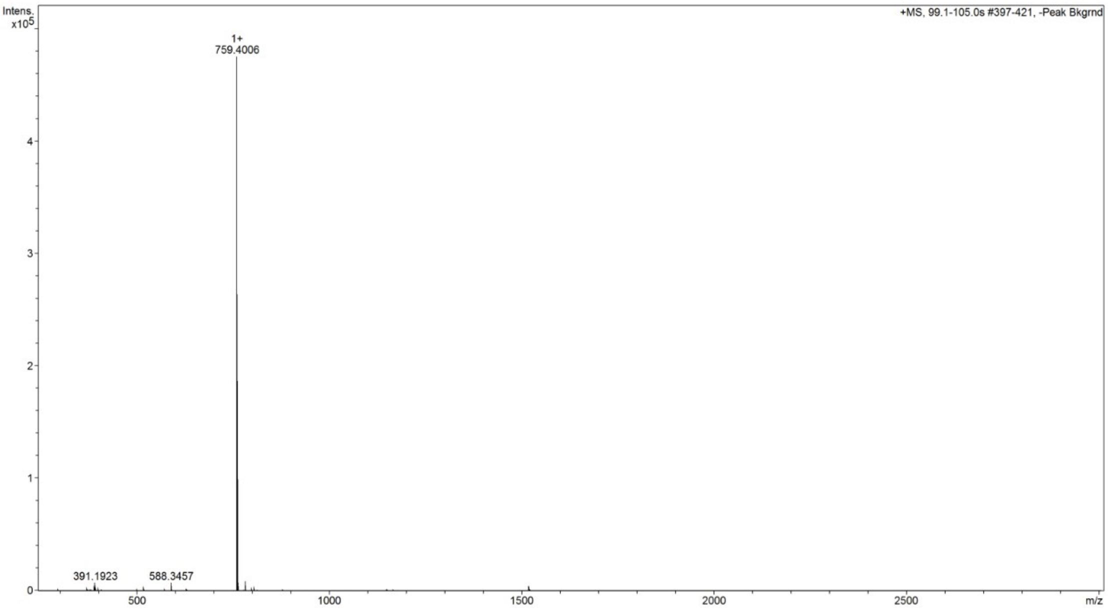

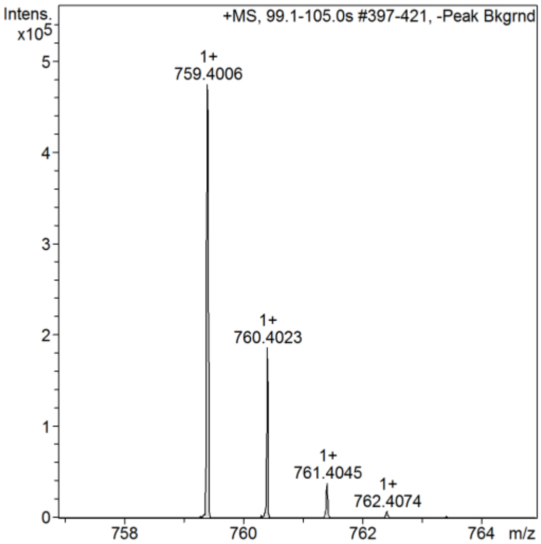

2.2 HPLC analytical purity data of the tested peptides

FAM-Ahx-ELFN1$_{579\text{-}594}$

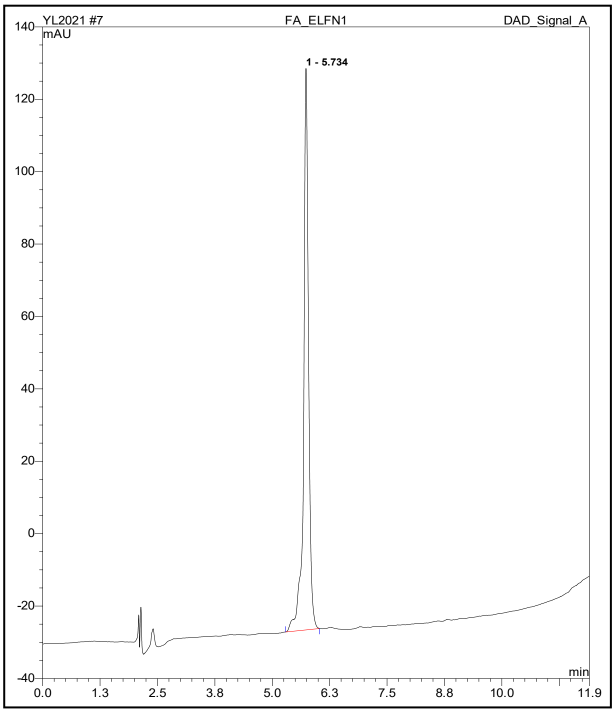

FAM-Ahx-GKAP-OH

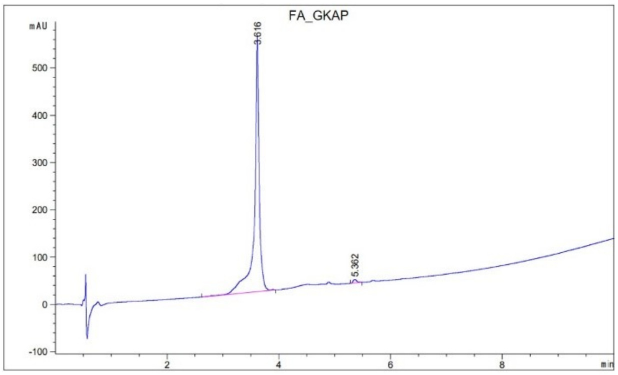

FAM-Ahx-ARAP3$_{1414\text{-}1429}$

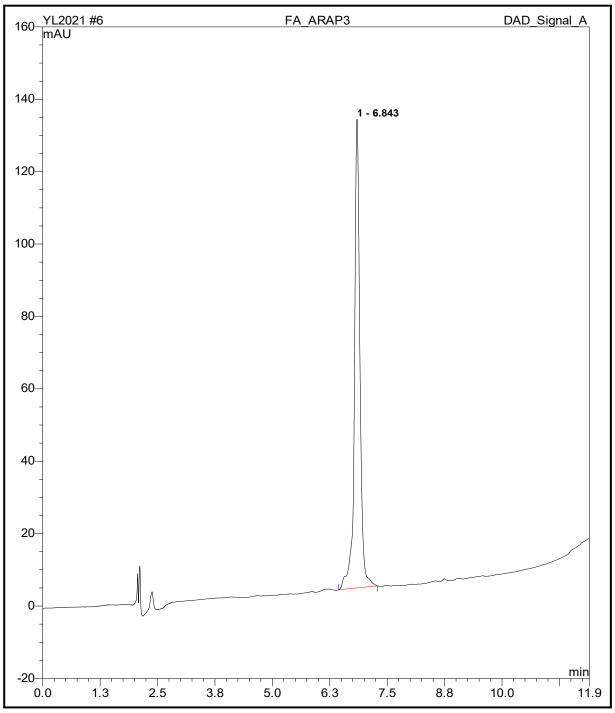

FAM-Ahx-EESTSFQGP-CONH$_2$

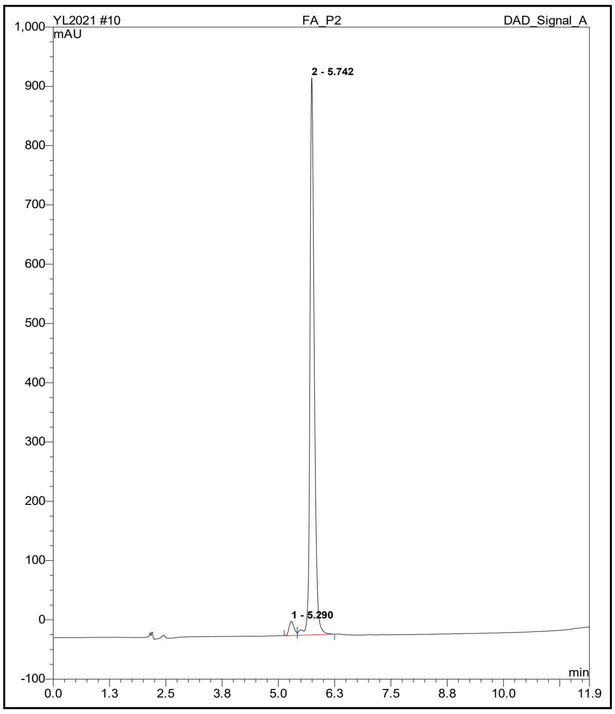

Ac-EESTSFQGP-CONH$_2$

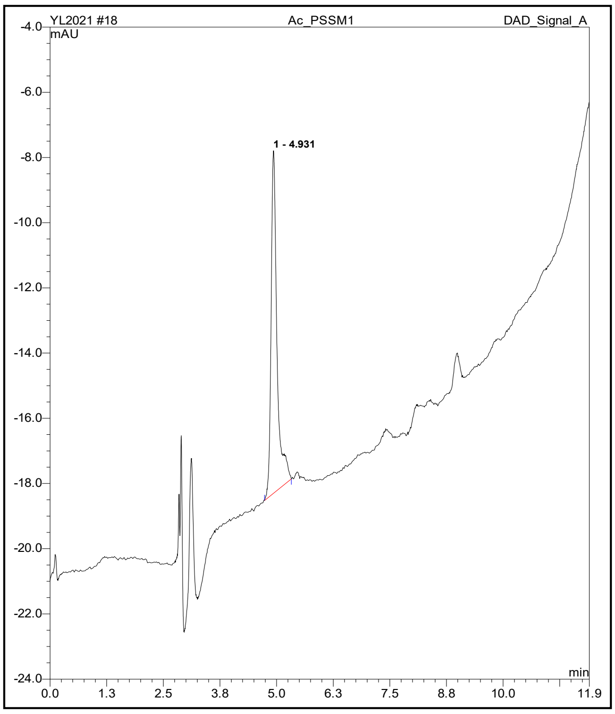

Ac-EAQTRL-CONH$_2$

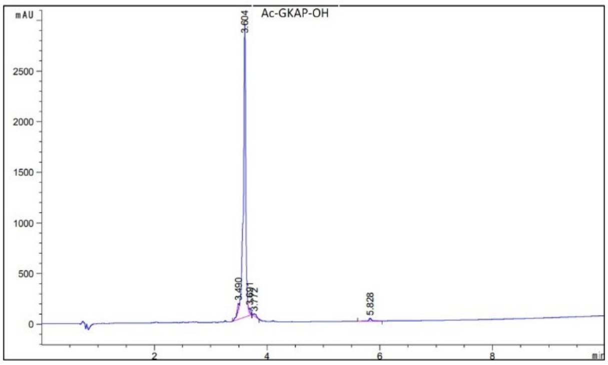

2.3 SDS-PAGE gel for the SHANK1 PDZ protein expression and purification

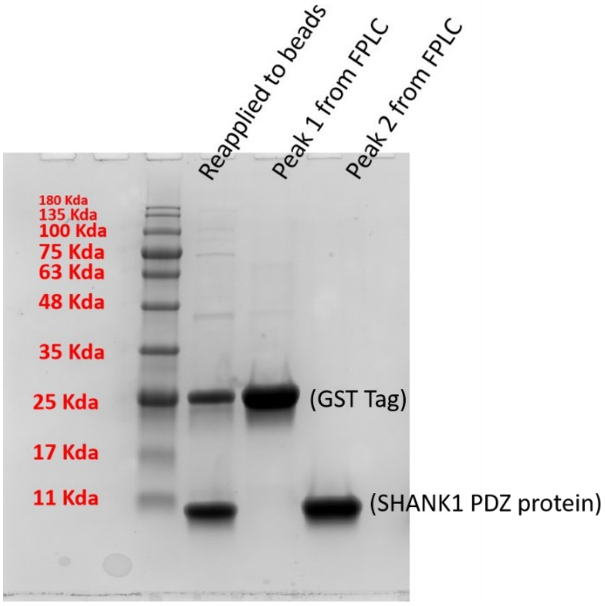

2.4 Mass spectrum of the SHANK1 PDZ protein expression

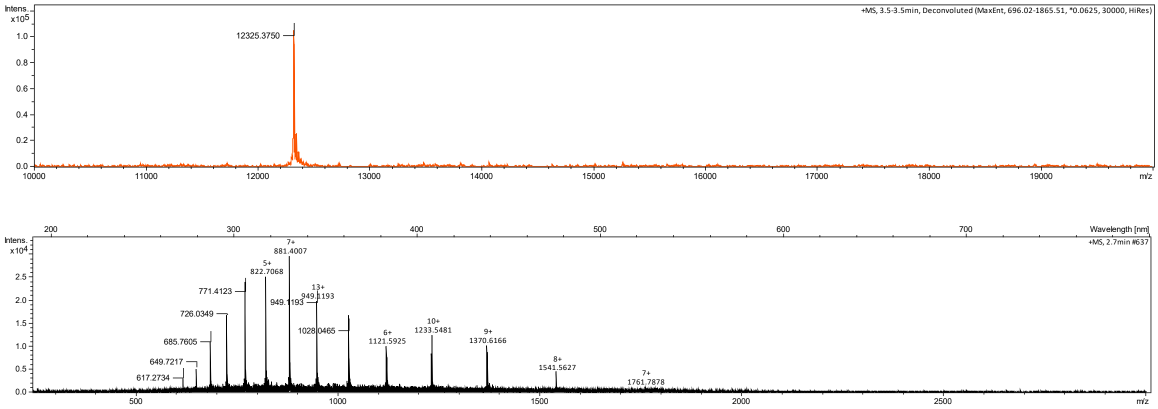

2.5 Chromatographic conditions for preparative HPLC$^{\alpha}$

Time (min)	Mobile phase A (%)	Mobile phase B (%)	Flow (mL/min)
0	90	10	15
20	50	40	15

Time (min)	Mobile phase A (%)	Mobile phase B (%)	Flow (mL/min)
25	20	80	15
30	0	100	15
33	0	100	15
35	90	10	15

$^{\alpha}$. Reversed Phase C18 column, mobile phase A (0.1% TFA in water), mobile phase B (0.1% TFA in ACN).

2.6 Equations for data calculation in fluorescent anisotropy direct titration

The intensity and anisotropy were calculated by using Equations 1 and 2. The data was fitted to a sigmoidal logistic model to obtain r_{min} and r_{max}, and the L_b was further calculated (Equation 3). Finally, the data was fitted to a nonlinear curve model (Equation 4), to obtain the value of K_D.

$$I = (2PG) + S \qquad \textit{Equation 1}$$

$$r = (S-PG)/I \qquad \textit{Equation 2}$$

$$L_b = (r - r_{min})/[\lambda(r_{max}-r) + (r-r_{min})] \qquad \textit{Equation 3}$$

$$y = \{(k+x+[FL]) - \sqrt{\{k+x+[FL]^2-4*[FL]\}}\}/2 \qquad \textit{Equation 4}$$

I = total intensity, r = anisotropy, P = perpendicular intensity, S = parallel intensity, G is an instrument gain factor, L_b = fraction ligand bound, $\lambda = I_{bound}/I_{unbound} = 1$, [FL] = concentration of fluorescent ligand, k = K_D and x = [added titrant].

2.7 Equations for data calculation in fluorescent anisotropy competition assays

The calculated average anisotropy values and their standard deviation were fitted using a sigmoidal logistic model (Equation 5).

$$y = r_{max} + (r_{min}-r_{max})/(1+(x/x_0)^p) \qquad \textit{Equation 5}$$

y = r = anisotropy, x_0 = mid-point of the curve between the r_{max} and r_{min} plateau.
